# The expression and function of HSV ICP47 and its promoter in mice

**DOI:** 10.1128/jvi.01107-23

**Published:** 2023-10-30

**Authors:** Thilaga Velusamy, Navneet Singh, Sarah Croft, Stewart Smith, David C. Tscharke

**Affiliations:** 1 John Curtin School of Medical Research, The Australian National University, Canberra, ACT, Australia; University of Virginia, Charlottesville, Virginia, USA

**Keywords:** herpes simplex virus, immune evasion, ICP47, latency, mouse model

## Abstract

Herpes simplex virus (HSV) protein immediate early-infected cell protein (ICP)47 inhibits the transporter associated with antigen presentation, thereby concealing infected cells from the CD8^+^ T-cell response in humans. By contrast, in mouse cells, ICP47 was found to be ineffective *in vitro*, despite some evidence for a role of this protein *in vivo*, specifically in the nervous system. Recent evidence suggests that the promoter that drives ICP47 might be active in the establishment of latency and during latency, leading to the possibility that a role might be found in protecting neurons that are latently infected with HSV from T-cell attack. Here, we explored this idea by studying a set of recombinant HSVs that were null for ICP47 protein and/or express Cre recombinase from ICP47 promoters, for use in a Cre-marking mouse model. Using these viruses, we found that contrary to previous studies, the activity of ICP47 in mouse cells is efficient enough to detectably reduce antigen presentation, albeit at levels much lower than in human cells. However, *in vivo*, we found no evidence of ICP47 influencing HSV pathogenesis or latency in a model where the virus does not spread beyond the peripheral nervous system. Finally, we show that the ability of the ICP47 promoter to drive protein expression during HSV latency is dependent on the location that it is placed in a recombinant virus genome and that such expression is undetectable from its native locus.

**IMPORTANCE:**

Immune evasion and latency are key mechanisms that underlie the success of herpesviruses. In each case, interactions between viral and host proteins are required and due to co-evolution, not all mechanisms are preserved across host species, even if infection is possible. This is highlighted by the herpes simplex virus (HSV) protein immediate early-infected cell protein (ICP)47, which inhibits the detection of infected cells by killer T cells and acts with high efficiency in humans, but poorly, if at all in mouse cells. Here, we show that ICP47 retains modest but detectable function in mouse cells, but in an *in vivo* model we found no role during acute infection or latency. We also explored the activity of the ICP47 promoter, finding that it could be active during latency, but this was dependent on genome location. These results are important to interpret HSV pathogenesis work done in mice.

## INTRODUCTION

Primary infection with Herpes simplex virus type 1 (HSV-1) begins at the local skin or mucosal surface where initial replication takes place in the epithelial cells. The virus quickly spreads to the nerve endings of neurons that make up the sensory or autonomous nervous system and further replication takes place in the cell bodies of these neurons. This productive or lytic phase is characterized by the expression of viral genes in the form of a cascade as immediate early (IE), early (E), and late (L) in the infected cells (1, 2). The lytic phase eventually subsides largely due to the engagement of an adaptive immune response, in particular CD8^+^ T cells. The viral genome persists in the surviving neurons as a latent reservoir with the ability to reactivate and cause productive infection or disease. During latency, transcription of lytic genes is limited and potentially restricted to some neurons or neuronal subtypes [reviewed in reference (3)]. Rather, latency-associated transcripts (LATs), a class of non-coding RNAs, are abundantly expressed in almost one-third of infected neurons at a given time (4 – 8).

The decline of the lytic phase coincides with an influx of virus-specific CD8^+^ T cells in the sensory ganglia of mice, which limits the spread of virus in an unconventional non-cytolytic manner (9 – 11). HSV-1 has evolved an efficient strategy driven by IE-infected cell protein (ICP)47 to evade CD8^+^ T-cell recognition of infected cells. ICP47 protein binds the peptide-binding site of the transporter associated with antigen processing (TAP), freezing its conformation and thereby preventing the transport and loading of antigenic peptides onto class I major histocompatibility complex-I (MHC-I) in the endoplasmic reticulum (ER) lumen (12 – 15). MHC-I is retained in the ER or Golgi apparatus without high-affinity peptides, so surface MHC-I levels are reduced, and viral peptides are not presented (12). This holds true for HSV-1-infected human fibroblasts that are resistant to the cytolytic effect of CD8^+^ T cells but is not the case for mouse fibroblasts (12). In further mechanistic *in vitro* studies, it was found that ICP47 is able to reduce the transport of peptides by TAP in human but not mouse cells (16, 17). Despite the absence of a function in mouse cells *in vitro*, ICP47 has been associated with increased neurovirulence, specifically the invasion of the virus into the central nervous system. Mice infected through a corneal route with an ICP47-deleted virulent strain of HSV-1, strain F, displayed little to no neurologic disease symptoms, whereas 60%–70% of mice succumbed to infection with wild-type or revertant virus (18). Whilst CD8^+^ T cells play an essential part in inhibiting viral replication in the cornea (19), the absence of ICP47 did not impact virulence at the periphery in the corneal epithelial cells (18). Another group revisited the functional relevance of ICP47 in an *in vivo* model, where mice were infected with a similar ICP47-deleted virus via a hematogenous route and again found that ICP47 enhances neuroinvasion (20). In this work, the role of ICP47 was found to be dependent on the presence of TAP in infected mice, suggesting again that the function of this viral protein is restricted to the inhibition of antigen presentation (20). Although both studies demonstrated that ICP47 does function *in vivo* in mice, the use of a lethal encephalitis model precluded any conclusions about the role of ICP47 in HSV-1 latency or reactivation.

HSV-1 latency in mice is dormant at an organismal level due to the absence of any peripheral shedding or reactivation events. However, low-level viral activity is common at the neuronal level, characterized by the presence of lytic transcripts along with latency-shaping LATs (reviewed in reference 3). Of relevance, ICP47 transcripts have also been found in latently infected sensory dorsal root ganglia (DRG) of mice inoculated with HSV on the flank (21). Evidence that lytic protein expression during latency is also possible came from the use of ROSA26 reporter mice, which have the potential to encode *lacZ* product beta-galactosidase (β-gal) following Cre-recombination at the ROSA locus (22). When these mice were infected with a recombinant HSV-1-expressing Cre from an ICP47 promoter, it was found that the number of marked cells continued to increase beyond the acute infection and into latency (23). These data suggest that ICP47 might be expressed at some level during latency.

The promoter that drives ICP47 expression lies in one of the repeats that bracket the unique short segment of the HSV-1 genome. This means it has a counterpart in the other repeat that drives another gene (ICP22); however, for simplicity, we will refer to this as the ICP47 promoter, unless stated. Elements upstream of the ICP47 coding sequence consist of the promoter as well as an intron, the former containing one of the HSV-1 origins of replication (OriS) and the transcription start site (Fig. S1) (24 – 29). The complexity of this region has been highlighted by several studies. Elements within the intron have been shown to negatively regulate the transcription of neighboring genes (30). Contrastingly, Greco and colleagues argued that elements downstream of the ICP47 promoter, excluding the intron, are required for efficient stimulation of the downstream gene (31). In addition, the removal of 62 bp to delete OriS reduced ICP47 promoter activity *in vitro*, but a recombinant virus made with this deletion had unaltered growth, pathogenesis, and reactivation (29). In this context, an important caveat of the Cre-marking study noted above is that the promoter sequences used were modified by removing the intron and the 62-bp OriS sequence (23). Furthermore, this promoter was part of a Cre-expression cassette that was then inserted ectopically in the HSV-1 genome in the space between genes U_L_3 and U_L_4. So, it remains to be shown whether expression from the native ICP47 promoter can be detected beyond the acute infection.

In this study, we characterized the role of ICP47 using a sensitive antigen presentation assay, and unlike past reports, we found that ICP47 retains detectable function in mouse cells *in vitro*. However, we were unable to find a phenotype for HSV-1 lacking ICP47 in a flank model of infection and latency. In addition, while being able to repeat previously published results showing that the ICP47 promoter can be active during latency, this was not the case when using the native promoter in its original location in the genome.

## RESULTS

### ICP47 can inhibit antigen presentation in mouse cells *in vitro*

**Fig 1:**
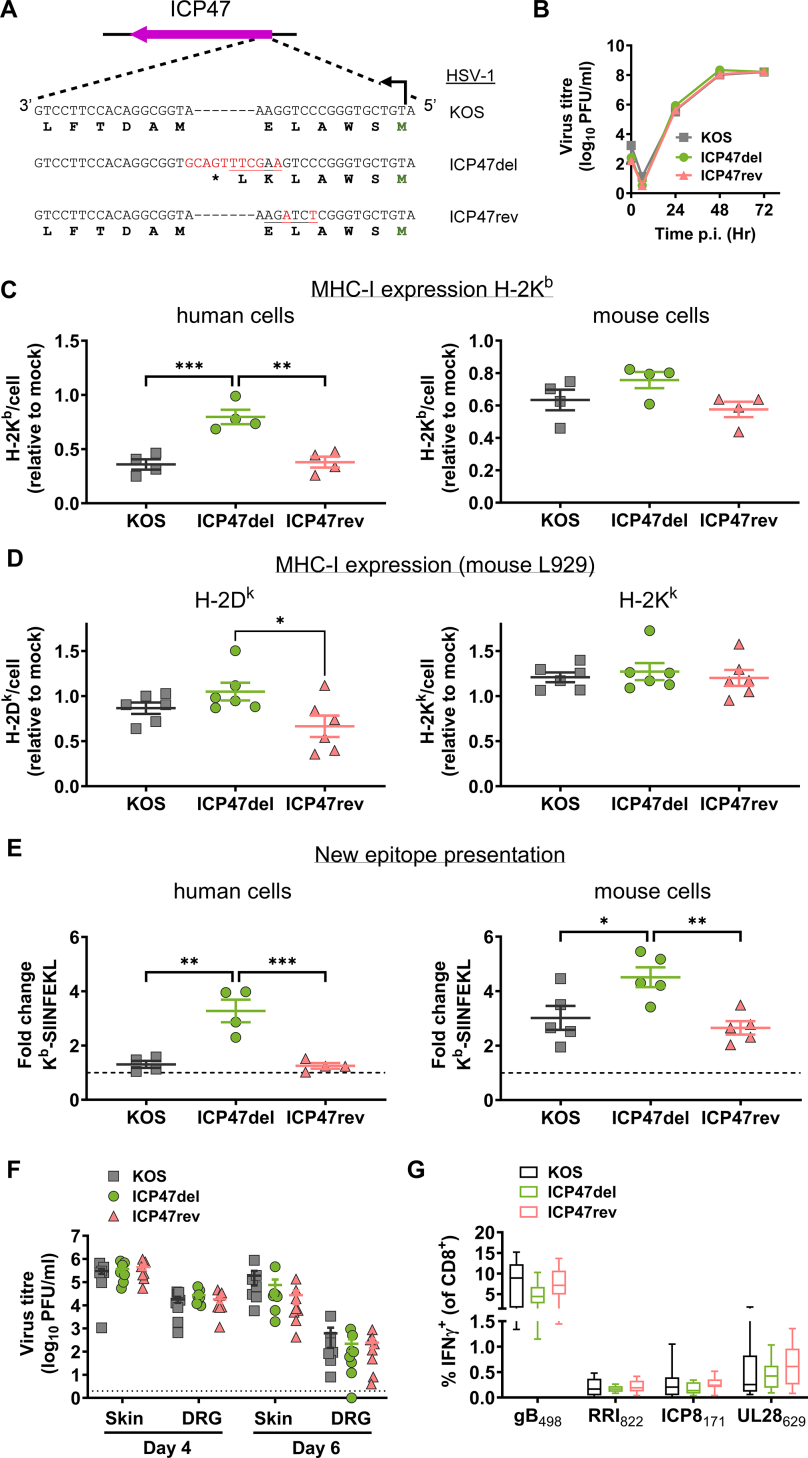
Function of ICP47 in mouse cells *in vitro* and *in vivo*. (**A**) Schematic showing nucleotide modifications (in red) in HSV-1 ICP47del and ICP47rev. Initiating codons (green) and stop (*) are marked, as are *Hind*III and *Xba*I enzyme sites (underlined) used to identify the recombinants. (**B**) Replication of recombinants assessed in Vero cells in comparison to parent virus. Data are shown as mean ± SEM from three replicates (error bars are obscured by data points). (**C**) Surface MHC-I (H-2K^b^) levels were quantified on 293KbC2 (left) and MC57G (right) cells after infection with HSV-1 recombinants or parent. Data are H-2K^b^ level relative to mock-infected samples from four independent experiments. (**D**) Surface MHC-I (H-2K^k^ and H-2D^k^) levels were quantified on L929 cells after infection with HSV-1 recombinants or parent. Data are H-2K^k^ or H-2D^k^ level relative to mock-infected samples from two independent experiments. (**E**) 293KbC2 (left) and MC57G (right) cells were infected with HSV-1 recombinants or parent, then with SIINFEKL-expressing M-miniOVA_257_, or M-miniFlu (as negative control). Data are the fold change in the levels of H-2K^b^-SIINFEKL relative to the negative control, and the dashed line represents the limit of detection. (**F and G**) Mice were infected on the flank with HSV-1 parent or recombinants by tattoo and viral loads (**F**) in the skin and DRG measured after 4 and 6 days, and CD8^+^ T cell responses (**G**) in spleen were measured. (**F**) Data are virus titers in an individual mouse derived from two independent experiments, and the dotted line signifies the limit of detection. (**G**) The percentage of CD8^+^ cells expressing IFNγ^+^ after stimulation with the HSV-1 peptides as marked is shown. Data are pooled from four independent experiments, each with three mice per group. The ends of boxes signify the interquartile range, the line shows the median, and the whiskers indicate maximum and minimum values. All panels unless specified: bars show mean ± SEM and statistical significance was determined using one-way ANOVA with Tukey’s multiple-comparison test (*P < 0.05; **P < 0.01; ***P < 0.001; and unmarked, not significant).

To investigate the role of ICP47 in HSV infection, we generated a set of ICP47-null and -revertant HSV-1 based on HSV-1 KOS. To generate the ICP47 null mutant (HSV-1 ICP47del) with minimal genetic changes, a stop codon was introduced downstream of the translation initiation site, preventing translation of the protein beyond the sixth amino acid (Fig. 1A). For ease of verification, a *Hind*III restriction site was also included, 3′ to the introduced stop codon. Additionally, as a control, a revertant virus (HSV-1 ICP47rev) was made by removing the stop codon from HSV-1 ICP47del and altering the restriction enzyme site to *Xba*I (Fig. 1A). These viruses showed similar growth *in vitro* as the parent (Fig. 1B). ICP47 reduces surface MHC-I indirectly by blocking peptide transport to cell surface (12, 13, 15), so we decided to functionally validate these viruses by quantifying MHC-I molecules on human-derived 293KbC2 cells (32). These cells are a stable transfectant that expresses a mouse MHC-I allele, called H-2K^b^, where the gene name is H-2K and the allele designation is b. This MHC-I molecule is expressed in C57BL/6 mice and allows a direct comparison of the efficiency of ICP47 in human and mouse cells. As expected, a reduction in MHC-I on infected cells relative to mock was seen for WT HSV-1 and the revertant but not ICP47del (Fig. 1C). The experiment was repeated using the mouse fibrosarcoma line MC57G and while there was no significant difference in MHC-I levels between parent, revertant, and ICP47del, we noted that the mean level of MHC-I was higher on cells infected with the ICP47-null virus than on either of the control viruses (parental KOS or ICP47rev) (Fig. 1C). Furthermore, when the data from parent and revertant were combined to make a single ICP47+ group, MHC-I was significantly reduced compared with ICP47del (*P* = 0.04; two-tailed *t*-test). This is the first time that a potential reduction of MHC-I expression due to ICP47 has been seen in mouse fibroblasts, so to extend the result, we repeated the previous experiments, but this time using L929 cells, which are derived from a C3H/An mouse and are of the H-2^k^ haplotype (H-2D^k^ and H-2K^k^). Infecting L929 cells with KOS and ICP47del resulted in no statistically significantly different H-2D^k^ levels on the surface of cells, though the mean level was higher where ICP47 was absent. Infection of these cells with ICP47Rev (a revertant that expresses ICP47) led to a significant reduction of H-2D^k^ levels compared with infections with ICP47del, but not wild-type KOS (Fig. 1D, left). Using the same approach as above and pooling data from KOS and ICP47Rev infections to compare a single ICP47+ group with the ICP47del group, the difference in H-2K^d^ was significantly different (*P* = 0.033; two-tailed *t*-test). Levels of H-2K^k^ were not significantly different on L929 cells infected with ICP47del compared with either KOS or ICP47Rev, individually or as a group. However, we noted again that the mean level of H-2K^k^ was slightly higher on cells infected with ICP47del, consistent with a trend across three MHC alleles and two cell lines.

Next, we turned to a more sensitive and, importantly, more relevant test of ICP47 function by investigating the ability of our viruses to block the presentation (on MHC-I) of a virally encoded peptide introduced at the same time as HSV-1 infection. To do this, cells were infected with the ICP47 deletion series of HSV-1 described above and then superinfected with a Modified Vaccinia virus Ankara (MVA) strain that expresses SIINFEKL peptide (M-miniOVA_257_) (33). The presentation of H-2K^b^-SIINFEKL complexes on these cells was measured by staining with a monoclonal antibody (25D1.16) and flow cytometry. As a negative control for SIINFEKL presentation, we used a similar MVA that expresses an irrelevant peptide (M-miniFlu). We found that human cells infected with the WT and revertant HSV had greatly reduced H-2K^b^-SIINFEKL presentation, compared with those infected with HSV-1 ICP47del (Fig. 1E). Surprisingly, a significant reduction in the presentation was also seen on mouse cells, though to a reduced extent than in the human cells. This is the first time that the inhibition of antigen presentation by ICP47 has been quantified directly on infected cells and the first demonstration that ICP47 retains some measurable function in mouse cells *in vitro*. While it would have been ideal to repeat these experiments with another peptide and MHC allele, reagents for such an experiment are not available. Taken together with the small reduction in total MHC-I after infection with expressing ICP47, we conclude that this viral protein is able to measurably reduce antigen presentation on MHC-I during HSV infection. These data provide a link between the original biochemical and *in vitro* work that suggested mouse TAP function was not affected by ICP47 and later experiments that found a phenotype for ICP47-deleted HSV in mice *in vivo*.

### Deletion of ICP47 from strain KOS does not alter virus growth or T-cell responses *in vivo*

Having shown an effect of ICP47 in mouse cells *in vitro*, we next examined the role of ICP47 *in vivo*. Previously, the role of ICP47 has been examined in mice infected by the ocular or intravenous routes with strains capable of neuroinvasion to the central nervous system. In the flank infection model using KOS, the virus is contained in the skin and peripheral nervous system, which is more typical of the average natural infection. C57BL/6 mice were tattoo-infected on the flank with ICP47del, ICP47rev, or parent KOS, and skin and innervating DRG were harvested on days 4 and 6 post-infection. The amount of virus in these tissues was similar irrespective of ICP47 expression on both days indicating that this gene does not play a role in HSV-1 replication in this model (Fig. 1F). We also examined CD8^+^ T cell responses after acute infection. The priming of CD8^+^ T cells during HSV-1 infection is thought to be largely by cross-presentation, in which uninfected dendritic cells take up and present virus antigens (34, 35). This suggests that ICP47 is unlikely to have an effect because it is confined to infected cells. However, the studies of antigen presentation by HSV-1 only examined responses to a single HSV-1 epitope (namely gB_498_; SSIEFARL) and priming pathways can differ for different viral antigens (33). To measure CD8^+^ T-cell responses to a broader set of HSV-1 epitopes, mice were infected with the ICP47 deletion series of viruses, and after 7 days, splenocytes from these mice were restimulated with synthetic peptides representing four HSV-1 epitopes and then stained for CD8 and intracellular IFN-γ. The number of cells positive for both markers in these spleens represents the extent of priming and expansion of HSV-1 epitope-specific CD8^+^ T cells during infection. Consistent with all epitopes being cross-primed by uninfected dendritic cells, there was no difference in the magnitude of CD8^+^ T-cell responses to any epitope in mice infected with ICP47-deficient or competent viruses (Fig. 1G). These results suggest that if ICP47 has a role in mice, it will be to blunt the effectiveness of CD8^+^ T-cell attack on infected cells and not their initial priming, which has been noted previously for an inhibitor of TAP expressed from cowpox virus (36).

### Role of ICP47 in the establishment and maintenance of HSV-1 latency

**Fig 2:**
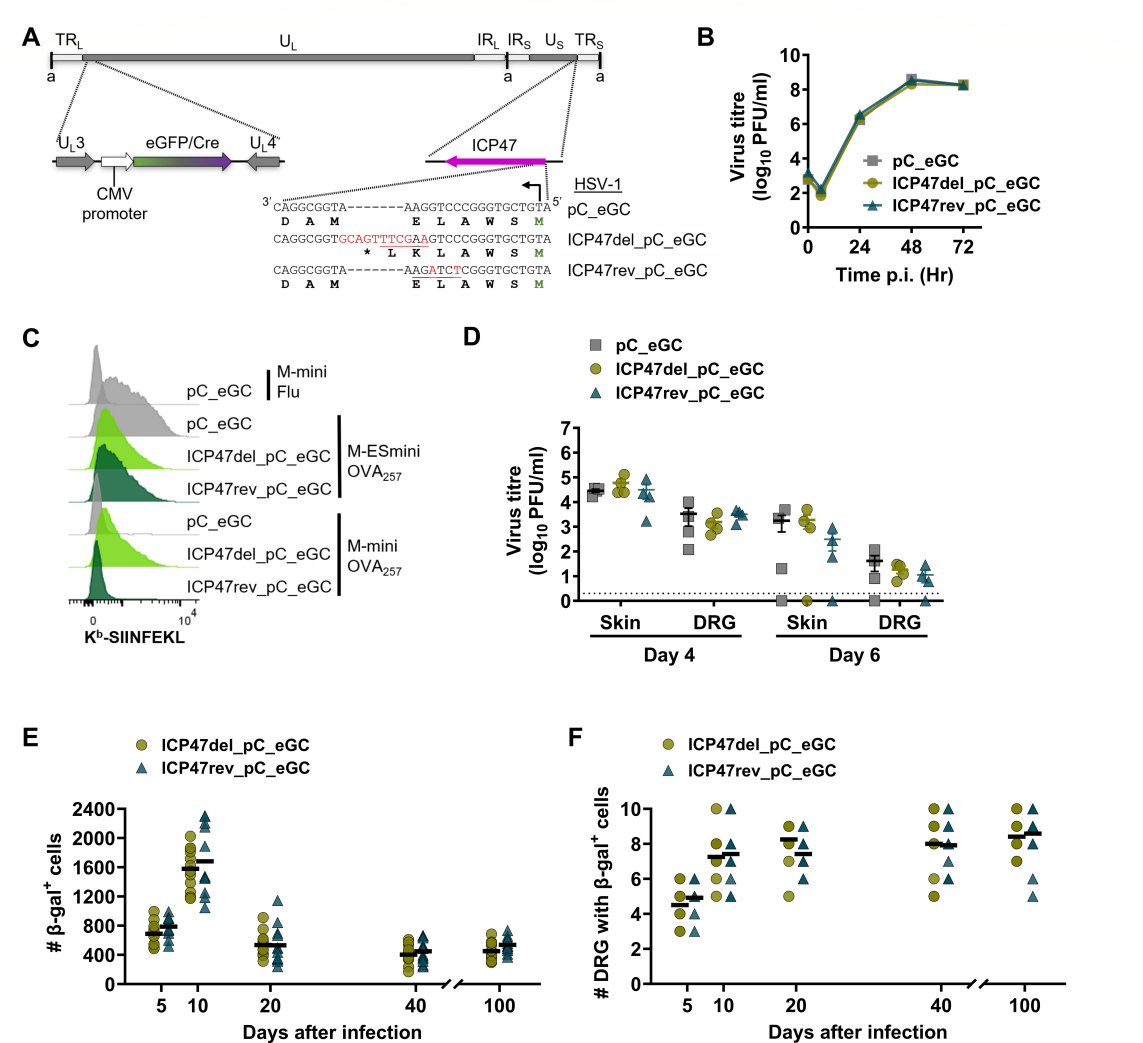
ICP47 does not play any role in the establishment and maintenance of latency. (**A**) Depiction of HSV-1 genome (to scale) showing unique long (U_L_) and short (U_S_) regions, short “a” repeats, and longer repeats (TR_L_/TR_S_; IR_L_/IR_S_). The U_L_3/U_L_4 region is expanded to show the eGFP/Cre (eGC) expression cassette, and nucleotide modifications in ICP47 are shown. (**B**) Replication of recombinant HSV-1 assessed in Vero cells in comparison to parent virus. Data are shown as mean ± SEM from three replicates (error bars are obscured by data points). (**C**) 293KbC2 cells were infected with HSV-1 recombinants or parent, then with SIINFEKL-expressing M-miniOVA_257_, M-ESminiOVA_257_, or M-miniFlu, and surface H-2K^b^-SIINFEKL measured by antibody staining and flow cytometry. (**D–F**) C57BL/6 (**D**) or ROSA26R (**E and F**) mice were infected on the flank with HSV-1 parent or recombinants by tattoo and viral loads (**D**) in the skin, DRG were measured after 4 and 6 days, and infected sites marked as β-gal^+^ (**E and F**) were counted at times shown. (**D**) Data are virus titers from an individual mouse, and the dotted line signifies the limit of detection. (**E and F**) The number of β-gal^+^ cells per mouse (**E**), and the number of DRG per mouse with at least one β-gal^+^ cell (**F**) from two independent experiments (overlapping values obscure total mouse number in panel **F**).

There is evidence that ICP47 might be expressed beyond the acute infection into the establishment phase of latency and maintained throughout latency (23). For this reason, we next sought to determine whether there was a role for ICP47 in the establishment or maintenance of latency using a ROSA26/Cre neuron-marking model to enumerate the number of latent neurons (23, 37 – 39). To do this, we constructed ICP47-null and revertant viruses as shown in Fig. 1, but on an HSV-1 background that expresses *eGFP/Cre* from the cytomegalovirus IE promoter in the U_L_3/U_L_4 intergenic region (Fig. 2A) (23). These ICP47-null and revertant viruses, named HSV-1 ICP47del_pC_eGC and ICP47rev_pC_eGC, respectively, showed no replication defects compared to parental HSV-1 pC_eGC in Vero cells (Fig. 2B). Next, we validated this new ICP47 deletion virus by detecting the presentation of H-2K^b^-SIINFEKL complexes on 293KbC2 cells co-infected with HSV-1 recombinants and MVA vaccinia viruses expressing various constructs that present SIINFEKL. In this case, as well as using M-miniOVA_257_ and a negative control (M-miniFlu), we used an MVA that inserts the SIINFEKL peptide directly into the ER, circumventing TAP (M-ESminiOVA_257_) as an addition control (33). We found that the level of SIINFEKL presentation was similar irrespective of the recombinant HSV-1 used on cells co-infected with M-ESminiOVA_257_, which allows the presentation to bypass TAP. By contrast, SIINFEKL presentation from M-miniOVA_257_ was inhibited by the parent and control HSV-1 but not the ICP47-deleted virus (Fig. 2C). To support the findings in the previous section, which found no role for ICP47 in acute HSV replication in mice, we used this new set of viruses in the flank infection model. The amount of virus in skin and DRG was similar across the three viruses on days 4 and 6 post-infection (Fig. 2D). This result is in line with that for our first ICP47del virus (Fig. 1F), confirming that ICP47 activity is not essential during acute infection of C57BL/6 mice. Importantly, this allows us to examine the ICP47 function and its promoter activity during the establishment and maintenance of latency knowing that interpretations will not be complicated by differences in initial virus replication and spread. To understand the effect of ICP47 on the fate of neurons, ROSA26 mice were tattoo-infected with HSV-1 ICP47del_pC_eGC or ICP47rev_pC_eGC on the flank. DRG were harvested and stained, and the number of β-gal-expressing neurons was counted. Consistent with the published results, the β-gal^+^ cell number increased from day 5 to 10, then decreased on day 20, and remained stable thereafter in latency (23). Furthermore, this pattern was the same for both viruses and similarly, the number of DRG with at least one β-gal^+^ cell, representative of the spread of the virus, was similar between ICP47-null and competent viruses (Fig. 2E and F). Altogether, we found no evidence that ICP47 plays a role in promoting the number of latently infected sites or protecting them, at any time after infection in C57BL/6 mice.

### No evidence for the activity of the native ICP47 promoter after acute infection

**Fig 3:**
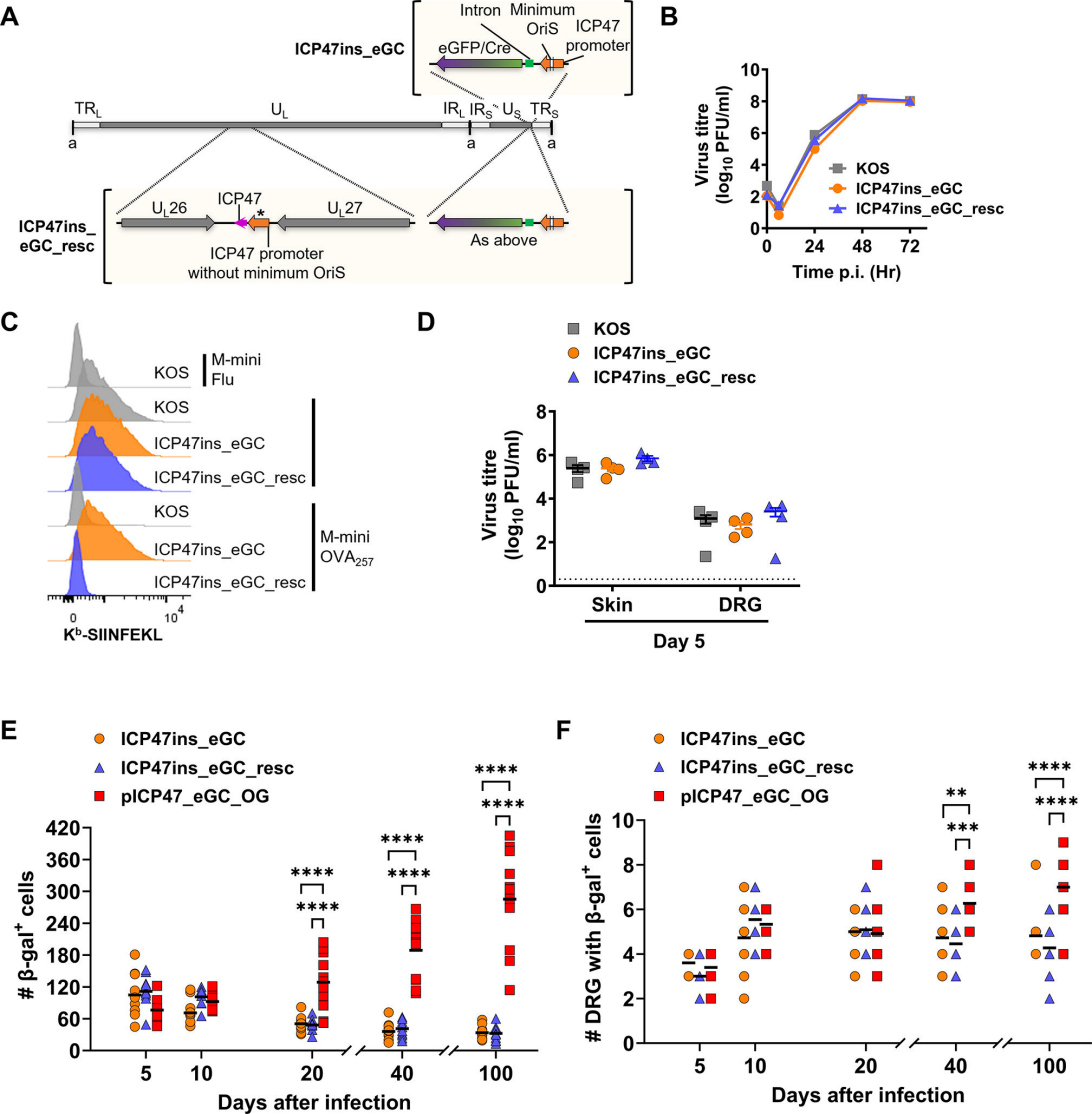
Evaluation of native ICP47 promoter activity *in vivo*. (**A**) Depiction of modifications made to generate HSV-1 genomes of ICP47ins_eGC (top) and ICP47ins_eGC_resc (bottom). Locations of eGFP/Cre and ICP47 in the genomes are shown. Asterisk indicates that an ICP47 promoter lacking OriS sequences was used. (**B**) Replication of recombinant HSV-1 assessed in Vero cells in comparison to parent virus. Data are shown as mean ± SEM from three replicates (error bars are obscured by data points). (**C**) 293KbC2 cells were infected with HSV-1 recombinants or parent, then with SIINFEKL-expressing M-miniOVA_257_, M- ESminiOVA_257_, or M-miniFlu, and surface H-2K^b^-SIINFEKL measured by antibody staining and flow cytometry. (**D–F**) C57BL/6 (**D**) or ROSA26R (**E and F**) mice were infected on the flank with HSV-1 parent or recombinants by tattoo and viral loads (**D**) in the skin, DRG were measured after 5 days, and infected sites marked as β-gal^+^ (**E and F**) were counted at times shown. (**D**) Data are virus titers from an individual mouse, and the dotted line signifies the limit of detection. (**E and F**) The number of β-gal^+^ cells per mouse (**E**) and the number of DRG per mouse with at least one β-gal^+^ cell (**F**) pooled from three independent experiments (overlapping values obscure total mouse number in panel **F**). Statistical significance was determined using ordinary two-way ANOVA with Tukey’s multiple-comparison test between viruses on a particular day (**P < 0.01).

Next, we moved from defining a role for ICP47 to examining the activity of its promoter with two further recombinant HSV-1 (Fig. 3A). In the first (HSV-1 ICP47ins_eGC), the coding sequence of ICP47 beyond the initiating methionine was replaced with *eGFP/Cre* so that Cre expression is under the control of native ICP47 promoter. The second (HSV-1 ICP47ins_eGC_resc) was made to restore ICP47 activity to ICP47ins_eGC by adding ICP47 under its own promoter into the intergenic space between U_L_26 and U_L_27. These new viruses grew at similar rates to HSV-1 KOS *in vitro* (Fig. 3B) and ICP47 function was absent and rescued in HSV-1 ICP47ins_eGC and ICP47ins_eGC_resc, respectively, as expected in our antigen presentation assay (Fig. 3C). Finally, virus loads after flank infection of mice were no different for these new viruses compared with the parent (Fig. 3D). Having validated these viruses, we used them to investigate if the native ICP47 promoter is active in latency by infecting ROSA26 mice on the flank and examining β-gal activity in DRG at various times after infection. We included the virus used by Russell and Tscharke (23) in these experiments, which has Cre expression under an ICP47 promoter between U_L_3 and 4 (called pICP47_eGC_OG here). We found that for viruses where *eGFP/Cre* was driven from the native promoter, the number of marked neurons fell from acute times to early latency and then remained similar. This was in stark contrast to previously published results that were repeated here for HSV-1 pICP47_eGC_OG, where the β-gal^+^ cells steadily increased on days 20, 40, and 100 (Fig. 3E). Additionally, the number of β-gal^+^ DRG increased for HSV-1 pICP47_eGC_OG from day 5 to later days, and on days 40 and 100, this was significantly higher compared with HSV-1 ICP47ins_eGC and ICP47ins_eGC_resc (Fig. 3F). Taken together, these experiments found evidence for the activity of the ICP47 promoter beyond acute HSV infection only when it was placed ectopically in the virus genome, between U_L_3 and 4, and not from its native location.

### Dissecting the role of ICP47 promoter elements

In reassessing the conflicting results regarding ICP47 promoter activity from the last experiment, we noted that there were two main differences between viruses that did and did not mark neurons after the acute infection: first, the location of the ICP47 promoter and associated expression cassette in the genome; and second, the structure of the ICP47 promoter. In the case of the viruses made for this paper, Cre was driven by the native promoter, which includes an OriS and has an intron between the end of the promoter and the start of the coding sequences. By contrast, HSV-1 pICP47_eGC has a modified ICP47 promoter, which has a 62-bp deletion that removes a part of OriS, is placed immediately upstream of the *eGFP/Cre* coding sequence, and this cassette is in the genome between U_L_3 and 4.

**Fig 4:**
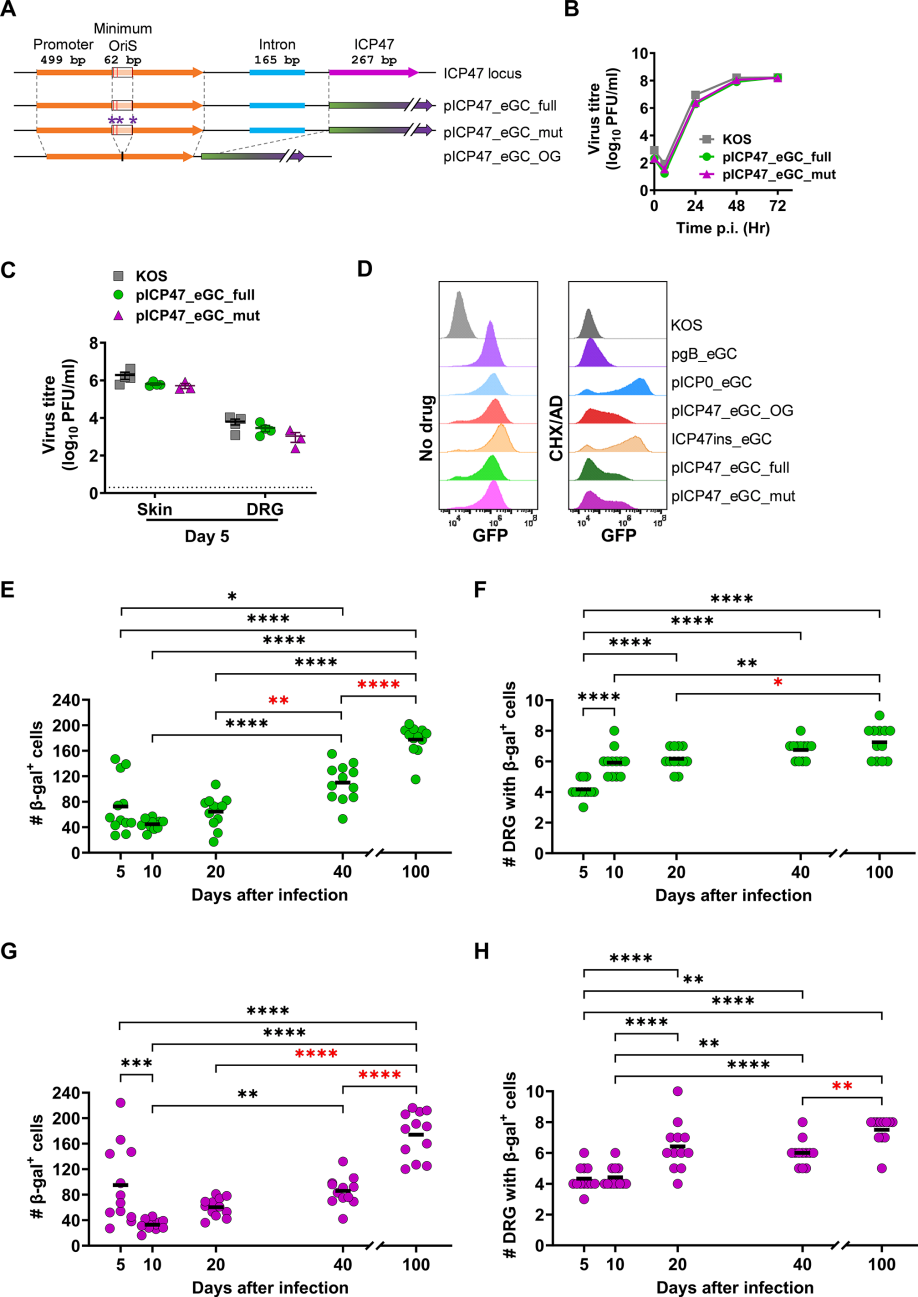
Design of ICP47 promoters and HSV-1 recombinants to investigate ICP47 promoter activity. (**A**) Schematic showing the features of the region upstream of the ICP47 open reading frame in the native locus (top) and as used in recombinant viruses, where it drives Cre from between U_L_3 and U_L_4. Deletions are shown with dotted lines and single bp changes are indicated with asterisks. (**B and C**) Replication of HSV-1 recombinants as marked *in vitro* (**B**) and *in vivo* (**C**), markers and symbols and statistical analyses are the same as in previous figures. (**D**) Promoter activity was measured based on GFP expression for recombinant viruses under control (no drug) or cycloheximide release conditions (CHX/AD) from the viruses as shown. (**E–H**) Groups of ROSA26 mice were infected with HSV-1 pICP47_eGC_full (**E and F**) or pICP47_eGC_mut (**G and H**) and DRG were taken and stained for β-gal expression at the days shown. The number of β-gal^+^ cells per mouse (**E and G**) and the number of DRG per mouse (**F and H**) are shown as in the previous figures. Data are pooled from three independent experiments with at least two overlapping time points. Differences between means were determined using a one-way ANOVA with Bonferroni’s multiple-comparison test (*P < 0.05; **P < 0.01; ***P < 0.001; ****P < 0.0001; and red stars signify differences in latency).

We chose to examine the promoter elements first and to do this, we made two further viruses in which variants of the ICP47 promoter were used to drive *eGFP/Cre* from between U_L_3 and 4 (Fig. 4A). The first used the full promoter sequence, with intron and OriS intact (pICP47_eGC_full). The second virus had three dinucleotide mutations in OriS to disrupt binding of the origin binding protein (U_L_9) but maintained the length of the natural promoter and the intron (pICP47_eGC_mut). Both viruses had growth *in vitro* and *in vivo*, which was comparable to HSV-1 KOS (Fig. 4B and C).

Next, the fidelity of the modified promoters to the IE kinetic class was assessed using a cycloheximide reversal assay described previously (40). HSV-1 KOS does not express GFP and was used as a negative control. HSV-1 pgB_eGC and pICP0_eGC were included as examples of leaky late and IE promoters, respectively, having been previously characterized (23). We then tested HSV-1 pICP47_eGC_OG, pICP47_eGC_full, pICP47_eGC_mut, and ICP47ins_eGC. GFP expression was observed from all the promoters in the untreated samples; however, only pICP0 and the various versions of the ICP47 promoters showed evidence of expression in the cycloheximide reversal conditions, consistent with an IE designation (Fig. 4D). However, of the viruses driving GFP from ICP47 promoters, only when GFP was expressed from the native start codon of ICP47 (i.e., in its authentic location) was the proportion of eGFP^+^ cells and brightness of expression comparable with that from the ICP0 promoter. These results suggest that even though ectopic ICP47 promoters drive some expression with IE characteristics, the level of this expression is reduced and also appears to vary more across the population of infected cells. This is consistent with lower-level luciferase expression driven from the same promoter with or without OriS expressed ectopically from the unique long segment (near the 3′ end of U_L_49) of HSV (29).

Finally, the expression of Cre from these promoters was investigated in our Cre-marking mouse model of infection. When ROSA26 mice were infected with pICP47_eGC_full, the number of β-gal^+^ cells was similar on days 5 and 10 with an apparent increase at day 20, which became statistically significant at day 40 and further increased again at day 100 (Fig. 4E). The number of DRG with at least one β-gal^+^ cell also increased over the course of the experiment, including from day 20 to day 100 (Fig. 4F). A similar pattern of neuronal marking was seen with pICP47_eGC_mut, with the exception that the number of β-gal^+^ cells decreased between days 5 and 10 before increasing at every successive time (Fig. 4G). There was a significant increase in marking between day 40 and day 100, consistent with the expression from this promoter during latency. This was echoed by increases being seen in the number of DRG with β-gal^+^ cells (Fig. 4H). These data are consistent with those previously published (23) and above for HSV-1 pICP47_eGC_OG. Therefore, we conclude that neither the OriS within nor the intron after the ICP47 promoter significantly alters its activity during latency.

### Location of the promoter in the genome determines activity in latency

**Fig 5:**
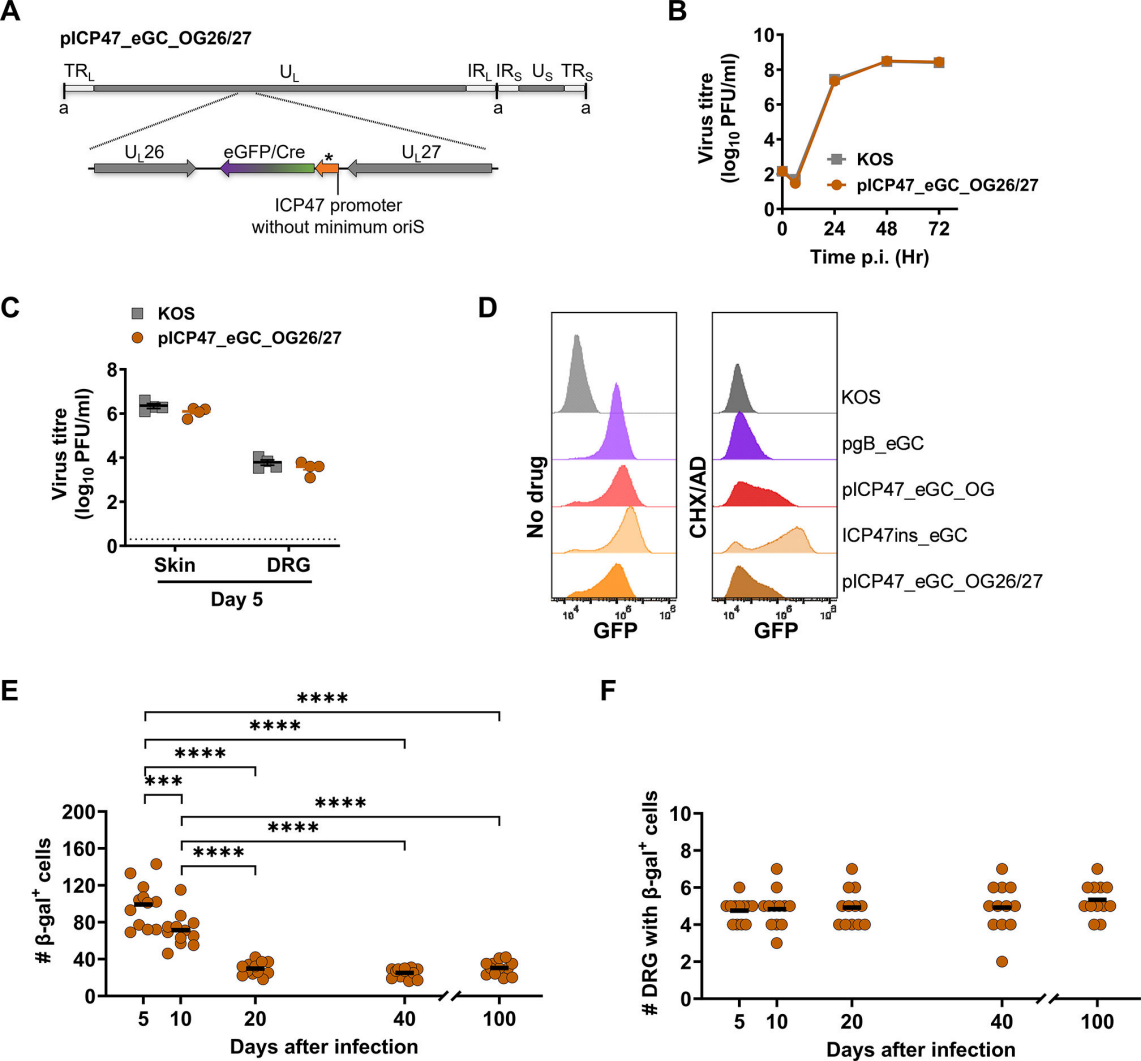
ICP47 promoter is not active in latency from the U_L_26/U_L_27 region. (**A**) Depiction of modifications made to generate HSV-1 genome of pICP47_eGC_OG26/27. Asterisk indicates that an ICP47 promoter lacking OriS sequences was used. (**B and C**) Replication of HSV-1 pICP47_eGC_OG26/27 *in vitro* (**B**) and *in vivo* (**C**), markers, symbols, and statistical analyses are the same as in previous figures. (**D**) Promoter activity was measured based on GFP expression for recombinant viruses under control (no drug) or cycloheximide release conditions (CHX/AD) from the viruses as shown. Data for control viruses are the same as in Fig. 4. (**E and F**) Groups of ROSA26 mice were infected with pICP47_eGC_OG26/27, and DRG were taken and stained for β-gal expression at the days shown. The number of β-gal^+^ cells per mouse (**E**) and the number of DRG per mouse (**F**) are shown as in previous figures. Data and statistical comparisons were as done in Fig. 4.

To check if the genome location of the ICP47 promoter influenced its activity in latency, we inserted the promoter-GFP-cre cassette from HSV-1 pICP47_eGC_OG in the intergenic space between U_L_26 and U_L_27 to make pICP47_eGC_OG26/27 (Fig. 5A). This recombinant virus was found to replicate to wild-type levels both *in vitro* and *in vivo* (Fig. 5B and C). To confirm if the ICP47 promoter belonged to the IE class when placed in the U_L_26/U_L_27 region, a cycloheximide release assay was done. This assay found that like the other ectopic ICP47 promoters described above, some expression was observed in cycloheximide release conditions, but this was not as strong or consistent as when expression was from the native locus (Fig. 5D). When ROSA26 mice were infected with this virus, the number of β-gal-producing cells decreased between days 5 and 10 and then even further to day 20 (Fig. 5E). In stark contrast to ICP47 promoter activity in latency from the U_L_3/U_L_4 region, but similar to the native locus, there was no further activity from ICP47 promoter in latency on days 40 and 100 when placed in U_L_26/U_L_27 region. Moreover, the number of DRG with β-gal^+^ cells remained similar throughout the infection (Fig. 5F). These results suggest that promoter activity in latency is dependent on the location of the promoter in the viral genome.

## DISCUSSION

Here, we aimed to answer two main questions related to HSV-1 ICP47 and its promoter in mouse models, which would have an impact on our understanding of the function of this viral immune evasion gene. First, we wanted to directly measure the impact of ICP47 expression on antigen presentation in mouse cells and compare this with its activity in human cells. Second, we wanted to ask whether activity from the ICP47 promoter could be detected during latency. To answer these questions, we engineered a series of HSV-1 recombinants and tested antigen presentation capacity *in vitro* and promoter activity *in vivo*.

Testing the role of ICP47 inhibition of antigen presentation in mouse and human cells *in vitro* revealed that viruses expressing ICP47 were able to decrease the surface presentation of newly synthesized antigen-MHC-I complexes in cells of murine origin. This is the first time that the direct activity of ICP47 has been able to be measured in mouse cells, though it is consistent with *in vivo* data that found a role of ICP47 in the neurovirulence of HSV-1 in mice (18, 20). The *in vivo* work noted above found that ICP47 played a role in promoting neuroinvasion of the virus into the CNS and encephalitis, but we were interested in testing a model where the virus and disease are restricted to theperipheral nervous system, which is more typical for human infections. In this setting, we found no decrease in viral loads associated with the deletion of ICP47 during acute infection in the skin or DRG. This is perhaps not unexpected because we used C57BL/6 mice and it has been shown previously that β2-microglobulin, and therefore by extension, CD8^+^ T cells are dispensable for control of HSV-1 disease in a flank model that used this mouse strain (41). However, we note that this earlier study did not quantify viral loads. We also looked at the number of latent sites established and the stability of those latent sites using a Cre-marking mouse model and again saw no differences when ICP47 was deleted from HSV-1. The main limitations of our experiments here, beyond the use of mice to model a human infection, are the strains of the virus, and probably more importantly, the mice we used. A significant role for CD8^+^ T cells has been shown in BALB/c mice, so it remains to be seen whether deletion of ICP47 from HSV-1 alters pathogenesis in these or other more susceptible strains of mice (10). A final result from this set of experiments that requires discussion is the lack of impact of ICP47 on the priming of CD8^+^ T cells in infected mice (Fig. 1G). This is most likely due to the intracellular function of ICP47 and the priming of CD8^+^ T cells during HSV-1 infection by uninfected dendritic cells (cross-priming) (35, 42, 43). However, we cannot rule out that some direct priming might occur and out-run the inhibition of TAP by ICP47, which has been shown for a similar inhibitor encoded by cowpox virus (33).

**Fig 6:**
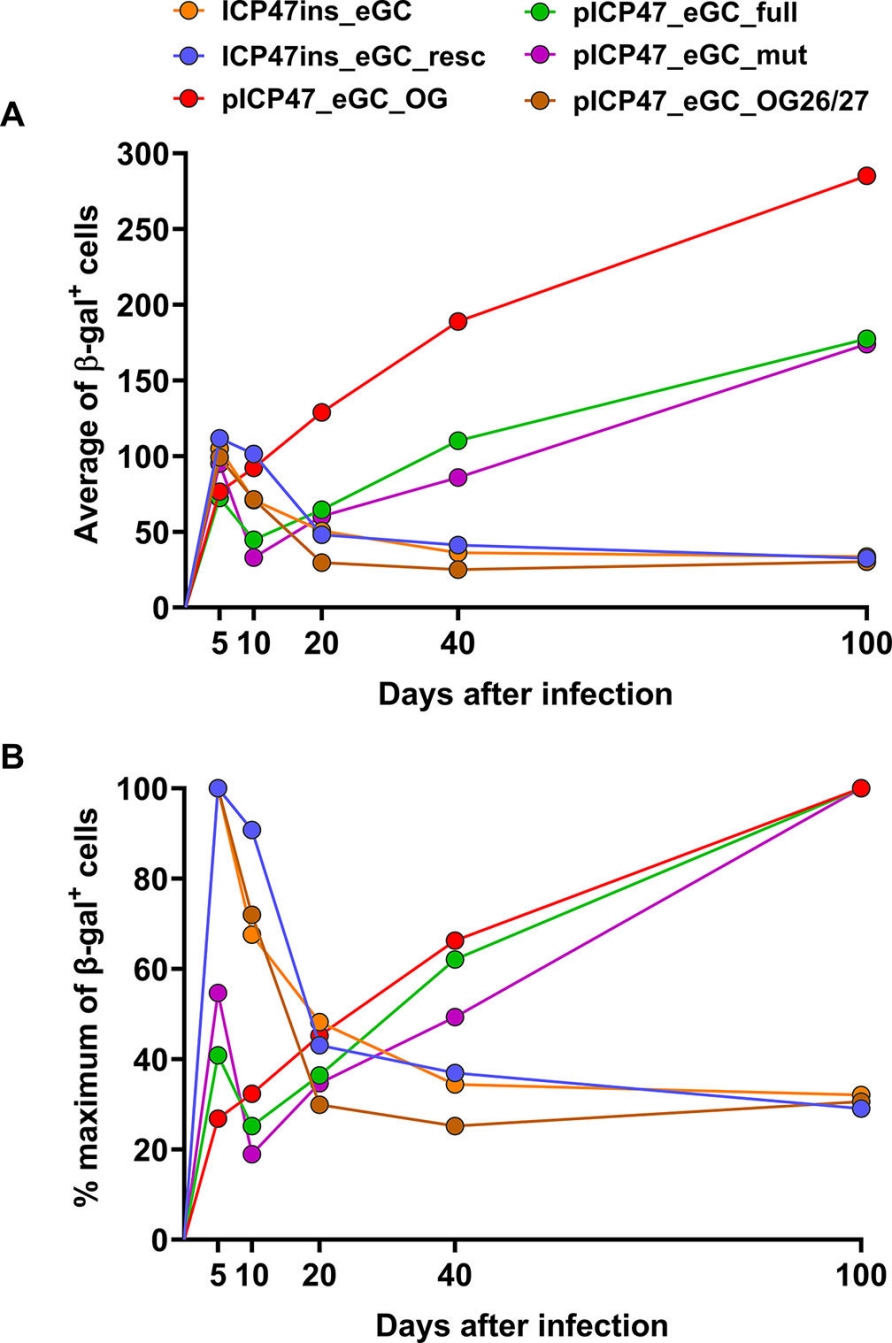
Summary of experiments where marked neurons were counted from HSV-1 ICP47 promoter recombinants. Marked neuron data is shown as the average number of β-gal^+^ cells (**A**) and the percentage of maximal value at the peak time point for each virus (**B**).

For our first experiments to examine the activity of the ICP47 promoter *in vivo*, we made a virus in which the ICP47 coding sequence was replaced with that of an eGFP-Cre fusion protein. When Cre-reporter mice were infected with this virus, we did not observe the continual buildup of marked neurons from the acute infection and into latency, which was seen previously when an ICP47 promoter was used to drive Cre from between U_L_3 and 4. This was confirmed in a side-by-side comparison of marking by these two viruses. We next aimed to identify the reasons for this difference. First, we investigated two sequences that were omitted from the promoter used in the published study, and second, the location of the promoter. In short, we found that any ICP47 promoter that was placed ectopically, irrespective of the sequences used, did not express with full authentic immediate early kinetics. This suggests that there are either elements missed further upstream in the mapping of this promoter or there are other features of the transcription unit, perhaps downstream of the open reading frame, which play a role. *In vivo*, we saw a different pattern across the viruses: it was only viruses in which the ICP47 promoter was placed between U_L_3 and 4 that could drive the expression of Cre during HSV-1 latency. This suggests that this region is uniquely permissive for expression during latency. Importantly, our study, which includes three different viruses with three different versions of the ICP47 promoter, shows that expression during latency from this location is a consistent finding and not an aberrant property of HSV-1 pICP47_eGC_OG. The neuron marking data from all of these viruses are presented as a summary, which shows absolute numbers as a comparison of intensity and also as a percentage of maximum marking for each virus to highlight the differences in kinetics or marking over time (Fig. 6).

In considering the new data here with that of previous studies, there are further points to note. Our work with the ICP47 promoter here might suggest that any HSV-1 promoter placed between U_L_3 and 4 would be permissive to latent transcription. However, in a previous study, the ICP0 promoter was not active during latency when placed in this region. This means it is likely that there are functional differences between promoters that result in different expression patterns in latency. Interestingly, previous data show that while the ICP0 promoter was not active between U_L_3 and 4, the gB (U_L_27) and the ICP6 (U_L_39) promoters did drive Cre expression beyond the acute infection from this region (23). Other studies have shown no activity in latency of the gC (U_L_44), ICP0, U_L_23, ICP4, and VP16 promoters when inserted into U_S_5 (38, 39). Together, our data and the previous studies suggest that U_S_5, U_S_12, and U_L_26/27 appear to be sites that are unfavorable for expression during latency. Conversely, U_L_3/4 is a site that supports gene expression in latency from a number of lytic gene promoters, pointing to a complex pattern of transcriptional regulation during latency.

Looking at the HSV-1 genome map, the most likely reason for the continued activity of promoters from U_L_3/4 in latent infection is the close proximity of this site to the LAT transcription unit. The region of the HSV-1 genome that encodes LATs is derepressed during latency, allowing these transcripts to be abundantly expressed in some neurons during latency (44). Just as is the case for host genes, the expression of HSV genes is dictated by chromatin modification, as reviewed recently (45, 46). During latency, the LAT region is enriched for typical active euchromatin histone modifications, whereas lytic promoters are enriched for repressive heterochromatin modifications. We speculate that the euchromatic modifications that permit LAT expression may extend further downstream, at least on some genomes, making the U_L_3/4 intergenic region partially permissive for transcription during latency. Having noted this, it is important to remember that the techniques used to examine the histone modification provide a global or bulk assessment across all infected cells, which might be misleading given the heterogeneous nature of HSV-1 latency. This heterogeneity may include differences in the level of repressive marking on individual HSV genomes.

The apparent permissiveness of transcription from the U_L_3/4 intergenic region during latency is interesting in the context of understanding the function of neighboring genes. The U_L_1 gene is located adjacent to the repeat-long (R_L_) region and upstream and in the opposite orientation to the LAT transcription start site. It has also been recently suggested that there is a LAT transcriptional region that extends from within the U_L_1 coding region to the LAT promoter (47). U_L_1 and U_L_2 have overlapping mRNA and potential shared poly(A) termination signal; however, this sequence is not strong. As a result, transcription starts with U_L_1 and runs on through the first terminator to the more efficient termination signal after U_L_3 (48). In this way, U_L_1–U_L_3 can be considered as a gene cluster in which at least some transcription begins from within a region associated with LAT and gene expression during latency. It is unclear what the placement of our expression cassettes into this region might do to disrupt this broader unit, but it might be that there may be a role for more permissive transcription of genes lying near the LAT region during latency. It would be of interest to know if the region on the other side of the unique long region, which has similar proximity to LAT, is also more permissive to gene expression during latency.

Three important limitations of our data and the above discussion need to be considered. First, we are inferring the behavior of modified HSV genomes based on what has been learnt from wild-type viruses, and the extent to which the addition of foreign DNA with different GC content and potentially other subtle characteristics is unknown. It will be informative to examine the exact histone profile of ectopic promoters placed within the U_L_3/4 intergenic region. Further along this line, it remains to be seen whether the native promoters in this region support gene expression during latency. Second, the above conclusions tacitly assume that Cre-reporter mouse models are capable of absolute sensitivity, in other words, that any Cre expression, no matter how low-level or brief, will be able to catalyze the recombination of *loxP* sites and lead to reporter gene expression. This assumption is demonstrably false based on studies of other models that have led to codon-modified Cre variants (49, 50). For this reason, a more conservative conclusion is that transcription during latency is more frequent and/or at a higher level from the U_L_3/4 region than from places in the genome further away from LAT. Transcription has been detected from across the genome in a high proportion of latent neurons (21), but the translated products of these transcripts might not be abundant enough to be detected by our Cre-marking mouse model. Improvements to Cre sensitivity may reveal more gene expression. Finally, all of this work is being done in mice and not in humans, the host species with which HSV-1 has co-evolved for millennia. From the first publications of ICP47, through our data here, if nothing else, this viral gene demonstrates that virus-host interactions can have exquisite species sensitivity.

## MATERIALS AND METHODS

### Mice

Eight-week-old, specific pathogen-free, female, C57BL/6 or B6.129S4-Gt(ROSA)26Sortm1So/J (ROSA26R) mice were obtained from the Australian Phenomics Facility, Canberra, ACT.

### Cell lines

Vero cells (CCL-81, ATCC) were maintained in Minimal Essential Media (MEM; Life Technologies) supplemented with either 2% or 10% heat-inactivated fetal bovine serum (FBS), 4 mM L-glutamine (Gibco), 5 mM HEPES buffer (Invitrogen), and 50 µM β-mercaptoethanol. HEK-293A (293A; CRL-1573, ATCC), HEK-293AKbC2 [293KbC2 (32)], MC57G (CRL-2295, ATCC), and L929 (NCTC clone) (85103115, CellBank Australia) cell lines were maintained in Dulbecco’s modified Eagle’s media (ThermoFisher) supplemented with 2% or 10% FBS and 2 mM L-glutamine. Cells were subcultured twice weekly.

### Viruses

All herpes simplex viruses were grown and titrated by plaque assay on Vero cells, as previously described (51). Parental HSV-1 KOS was a gift from Dr. F. R. Carbone, and all recombinant viruses were generated on the background HSV-1 strain KOS. All HSVs used throughout this study are described in Table 1.

**TABLE 1:** Description of HSV-1 used throughout this study

Name	ICP47 expression^*a*^	eGFP/Cre expression	Parent virus^*b*^	Source
HSV-1 KOS	Native	Nil		
HSV-1 pC_eGC	Native	Promoter: CMV IE Location: UL3/4 intergenic region		(51)
HSV-1 ICP47del	Nil	Nil	HSV-1 KOS	This study
HSV-1 ICP47rev	Native	Nil	HSV-1 ICP47del	This study
HSV-1 ICP47del_pC_eGC	Nil	Promoter: CMV IE Location: UL3/4 intergenic region	HSV-1 pC_eGC	This study
HSV-1 ICP47rev_pC_eGC	Native	Promoter: CMV IE Location: UL3/4 intergenic region	HSV-1 ICP47del_pC_eGC	This study
HSV-1 ICP47ins_eGC	Nil	Promoter: ICP47 native Location: US12	HSV-1 KOS	This study
HSV-1 ICP47ins_eGC_resc	From UL26/27 intergenic region	Promoter: ICP47 with deletion of the OriS Location: US12	HSV-1 ICP47ins_eGC	This study
HSV-1 pICP0_eGC	Native	Promoter: ICP0 Location: UL3/4 intergenic region		(23)
HSV-1 pICP47_eGC_OG	Native	Promoter: ICP47 with deleted OriS Location: UL3/4 intergenic region		(23)
HSV-1 pICP47_eGC_mut	Native	Promoter: ICP47 with disrupted UL9 binding Location: UL3/4	HSV-1 KOS	This study
HSV-1 pICP47_eGC_full	Native	Promoter: ICP47 native Location: UL3/4	HSV-1 KOS	This study
HSV-1 pICP47_eGC_OG26/27	Native	Promoter: ICP47 with deletion of the OriS Location: UL26/27	HSV-1 KOS	This study
HSV-1 pgB_eGC	Native	Promoter: gB Location: UL3/4 intergenic region		(23)

^*a*^ Native ICP47 expression refers to endogenous ICP47 expression from the U_S_12 gene region remaining unaltered.

^*b*^ Parent virus is listed for the viruses made for this study.

Vaccinia viruses used as antigen donors or controls in the *in vitro* antigen presentation assay are MVA recombinants and are previously described as follows: M-miniFlu, published as MVA-ESmini-PB1-F2 (52), M-ESminiOVA_257_ (33), and M-miniOVA_257_ (53).

### Generation and isolation of recombinant viruses

Recombinant HSVs were generated by transfection-infection and CRISPR/Cas9 targeting techniques, as previously described (51). Briefly, monolayers of 293A cells were transfected using Lipofectamine 2000 (Life Technologies) with equimolar amounts of the HDR template plasmid or a ssODN, and the CRISPR-Cas9 plasmid (px330 Addgene plasmid 42230) with an appropriate guide RNA. After 5 hours of transfection, the transfection mix was removed and cells were infected with 0.01 pfu/cell of parental HSV-1, and after 2 hours of adsorption, the inoculum was replaced with fresh medium. Recombination and infection proceeded for 72 hours before supernatants containing the released virus were collected and stored at −80°C. Thawed transfection/infection mixes were diluted fivefold and used to infect monolayers of Vero cells before viruses associated with single plaques were isolated and subject to PCR screening. Multiple rounds of growth and single plaque purification were conducted until stocks were free from parental virus. Genome modification and insertion sites were verified by Sanger sequencing and restriction fragment length polymorphism analysis.

### Viral replication analysis *in vitro*

Viral replication kinetics of newly generated viruses were tested alongside parental viruses to identify any replication deficits. Vero cells were infected with 0.01 pfu/cell of HSV-1 and after 1 hour of adsorption at 37°C, the inoculum was removed, the cells were washed twice with warm PBS, and MEM-2 was added to the wells. At indicated time points, both cell-associated and released viruses were collected by scraping cells and collecting them along with media. Collected samples were subject to three freeze-thaw cycles before determining the virus titer by standard plaque assay.

### Cycloheximide reversal assay

To determine the kinetic restriction of GFP under each promoter, a cycloheximide reversal assay was used. Vero cells were pretreated for 1 hour with cycloheximide (100 µg/mL in MEM-2) before removal of media and infection of cells with 5 pfu/cell of respective HSV in MEM-0 containing cycloheximide. After 1 hour of adsorption, the inoculum was removed and replaced with cycloheximide containing MEM-2%. After a further 6 hours, cycloheximide was washed out and replaced with media containing actinomycin D (5 µg/mL) before 4 hours of incubation. Cells were washed and collected before fixation and flow cytometric analysis for GFP expression. Controls were infected but remained untreated completely or were subject to treatment of either cycloheximide only or actinomycin D only.

### Verification of ICP47 activity *in vitro*

To verify the activity of ICP47 expressed from newly generated recombinant HSV, 293KbC2 or MC57G cells in suspension were infected with HSV-1 at an MOI of 10 pfu/cell in D0 or remained uninfected as control. Cells were incubated at 37°C with gentle agitation for 3 minutes and then shaken at 250 rpm for a further 30 minutes. Cells were diluted with warm D2 and incubated with rotation at 37°C for 2.5 hours. Cells were pelleted by centrifugation and resuspended in media containing 10 pfu/cell of “antigen donor viruses”: MVA-miniOVA, MVA-ESminiOVA, MVA-ESminiPB1-F2, or 10^−7^ mM SIINFEKL peptide before incubation with agitation, shaking, and rotation as described above. After 5.5 hours of rotation, cells were pelleted by centrifugation and washed before labeling the cells with H-2K^b^/SIINFEKL-specific antibody (clone 25D.1.16) for fixation and flow cytometry.

### Quantitative flow cytometry

To determine the expression of MHC-I molecules on cells infected with recombinant HSV-1, QIFIKIT quantitation beads (Dako) were used. Briefly, cells of interest were infected with HSV-1 at an MOI of 5 as described in the previous sections, for a total of 6 hours. Cells were collected by centrifugation and washed with PBS supplemented with 2% fetal bovine serum(FBS). Cells were stained with anti-H-2K^b^ [culture supernatant from the hybridoma HB176 (mAb also known as Y-3), which was a kind gift from J. Yewdell, NIAID, NIH, Bethesda, MD, USA (54)], H-2K^k^ (36-7-5 clone; BioLegend), or H-2K^d^ (SF1-1.1 clone; BioLegend) and washed prior to staining with saturating concentrations of the FITC-conjugated anti-mouse F(ab)_2_ fragments before paraformaldehyde fixation. The calibration beads, consisting of five bead types, each with a defined number of antibody binding sites, were washed and stained with FITC-conjugated antibody as per the manufacturer’s instructions. Cells and beads were washed well before flow cytometric analysis. Fluorescence data from the calibration beads were used to create a standard curve by relating the mean fluorescence intensity obtained to the antibody binding capacity. The standard curve was then used to determine the antibody binding capacity and subsequently quantitate the amount of MHC-I on cellular samples.

### Infection of mice by tattoo

To determine viral replication or Cre expression in mice, the virus was introduced by a tattoo of the left flank. Briefly, shader needles were dipped in inoculum containing 10^8^ pfu/mL of HSV-1 before tattooing an area of 0.5 cm^2^ on the shaved flank, while mice were sedated by Avertin anesthesia.

### Viral titer from *in vivo* infection

To determine viral replication *in vivo*, at the experimental time point indicated, a 0.8 cm^2^ region of skin at the point of initial infection was excised or DRG from spinal levels T5-L1 were collected in 800 µL of D2. Skin or DRG samples were frozen before homogenization with a tissue grinder before the determination of viral titer by standard plaque assay.

### Cre-beta galactosidase neuron marking

To determine the historic Cre expression from recombinant viruses, at the indicated experimental time point, DRG from spinal levels T5-L1 were excised and collected in glutaraldehyde/paraformaldehyde fixative. DRG were washed and stained with the β-galactosidase substrate, X-Gal (5-Bromo-4-chloro-3-indolyl β-D-galactopyranoside) in permeabilization solution (51), before washing and overnight glycerol clarification. Clarified DRG were mounted for microscopy and imaged with Olympus CKX41 light microscope and Olympus DP20 camera. Neurons containing the blue X-gal precipitate are indicative of cells that have had Cre expression from HSV-1 driven by the promoter of interest and were enumerated.

### Measurement of the CD8^+^ T-cell response

To determine the magnitude of the CD8^+^ T-cell response, peptide-specific CD8^+^ T cells that produce IFN-γ were quantitated from the spleens of mice 7 days post-infection, as previously described (55). Briefly, single-cell suspensions of 1.0 × 10^6^ cells were incubated with 0.1 µM of synthetic peptide (gB_498-505_; SSIEFARL, RRI_822-829_; QTFDFGRL, ICP8_171-178_; INNTFLHL, UL28_629-637_; YSVENVGLL, all synthesized by Mimotopes at >90% purity) for 1 hour at 37°C and 5% CO_2_. After which, Brefeldin A (Sigma) was added at a final concentration of 5 µg/mL, and cultures were incubated for a further 3 hours. Cells were centrifuged to remove peptide and washed before resuspension in anti-CD8α-PE antibody (clone 53-6.7). After staining, cells were washed before paraformaldehyde fixation and intracellular staining with anti-IFNγ-APC antibody (clone XMG1.2) diluted in FACS-PBS buffer (PBS with 2% FBS) with permeabilzation agent (0.25% saponin). After staining, cells were washed and analyzed by flow cytometry. At least 50,000 events were collected, and doublet discrimination and live cell discrimination were performed before CD8^+^ IFNγ^+^ T cells were quantitated.

### Statistical analysis

Statistical significance was determined by one- or two-way ANOVA when comparing more than two groups, or a *t*-test when comparing two groups with multiple comparison analyses described in figure legends. All statistical analyses were done in GraphPad Prism. Statistical significance was accepted at *P* < 0.05.

## SUPPLEMENTAL MATERIAL

Fig. S1 jvi.01107-23-s0001.pdf Detail of the ICP47 promoter region.

ASM does not own the copyrights to Supplemental Material that may be linked to, or accessed through, an article. The authors have granted ASM a non-exclusive, world-wide license to publish the Supplemental Material files. Please contact the corresponding author directly for reuse.
