# Supplementary material for: The expression and function of HSV ICP47 and its promoter in mice
Source: J Virol. 2023 Oct 30;97(11):e01107-23. doi: 10.1128/jvi.01107-23 (PMC10688380; doi:10.1128/jvi.01107-23)
Supplement: Fig. S1 — Detail of the ICP47 promoter region. [file jvi.01107-23-s0001.pdf]

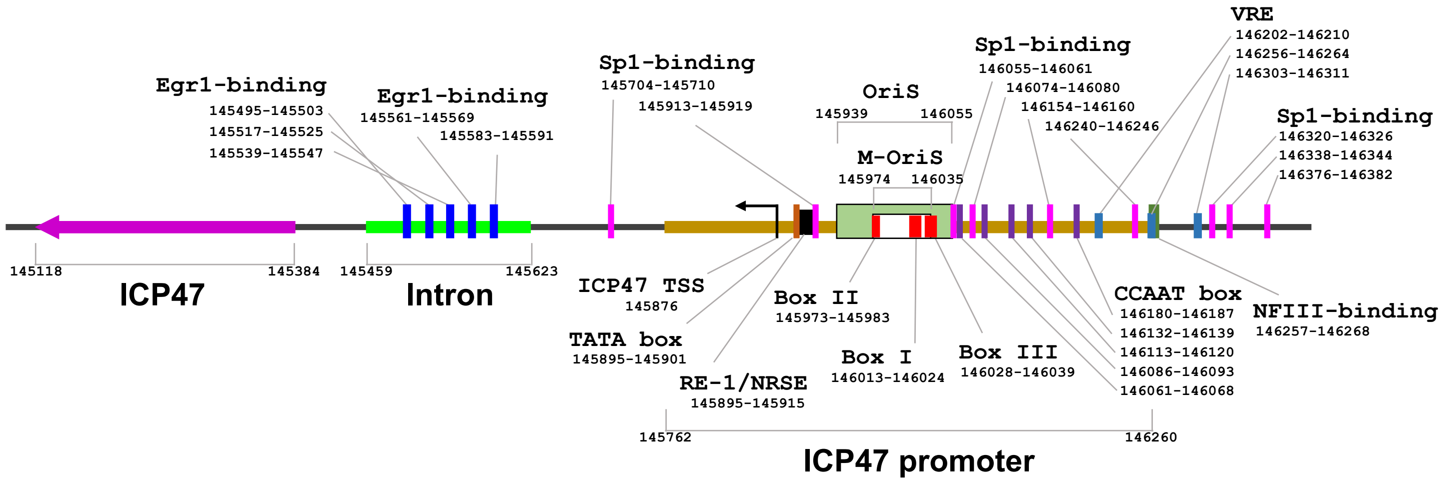

Supplementary figure 1: ICP47 promoter elements.

Schematic representation of ICP47 and the upstream region consisting of intron and ICP47 promoter (to scale). The region shown is from the U_S/TR_S junction of standard genomic isomer of HSV-1 and the base pair position is in reference to HSV-1 KOS genome (JQ673480). For each feature, the nucleotide number at the end indicates that the nucleotide at that position is inclusive. Origin of DNA synthesis within the promoter, OriS is shown containing a 62 bp sequence referred to as minimum oriS (M-OriS) (Summers and Leib 2002) and the U_L9-binding sites called Box I, II and III (Stow and McMonagle 1983, Elias and Lehman 1988, Koff and Tegtmeyer 1988, Olivo et al. 1988, Weir et al. 1989, Summers and Leib 2002). Other features present include binding sites for host-specific transcription factors such as Specificity protein 1 (Sp1; in pink), Early growth response protein 1 (Egr1; in dark blue) and NFIII (in forest green), and DNA elements such as VP16 responsiveness element (VRE; in light blue), TATA box (in brown), CCAAT box (in violet) and Restrictive Element-1/Neuronal Restrictive Silencer Element (RE-1/NRSE; in black). ICP47 transcription start site (TSS) is marked with an arrow.
